# Matrix analysis and risk management to avert depression and suicide among workers

**DOI:** 10.1186/1751-0759-4-15

**Published:** 2010-11-05

**Authors:** Takeaki Takeuchi

**Affiliations:** 1 Department of Hygiene and Public Health, Teikyo University School of Medicine, Tokyo, Japan; 2 Division of Psychosomatic Medicine, Teikyo University Hospital, Tokyo, Japan; 3 Affiliated hospital and Mental Health Center, Tokyo Gakugei University, Tokyo, Japan

## Abstract

Suicide is among the most tragic outcomes of all mental disorders, and the prevalence of suicide has risen dramatically during the last decade, particularly among workers. This paper reviews and proposes strategies to avert suicide and depression with regard to the mind body medicine equation hypothesis, metrics analysis of mental health problems from a public health and clinical medicine view.

In occupational fields, the mind body medicine hypothesis has to deal with working environment, working condition, and workers' health. These three factors chosen in this paper were based on the concept of risk control, called *San-kanri*, which has traditionally been used in Japanese companies, and the causation concepts of host, agent, and environment. Working environment and working condition were given special focus with regard to tackling suicide problems. Matrix analysis was conducted by dividing the problem of working conditions into nine cells: three prevention levels (primary, secondary, and tertiary) were proposed for each of the three factors of the mind body medicine hypothesis (working environment, working condition, and workers' health). After using these main strategies (mind body medicine analysis and matrix analysis) to tackle suicide problems, the paper talks about the versatility of case-method teaching, "*Hiyari-Hat *activity," routine inspections by professionals, risk assessment analysis, and mandatory health check-up focusing on sleep and depression. In the risk assessment analysis, an exact assessment model was suggested using a formula based on multiplication of the following three factors: (1) severity, (2) frequency, and (3) possibility.

Mental health problems, including suicide, are rather tricky to deal with because they involve evaluation of individual cases. The mind body medicine hypothesis and matrix analysis would be appropriate tactics for suicide prevention because they would help the evaluation of this issue as a tangible problem.

## Introduction

Suicide is among the most tragic outcomes of all mental disorders; among the G8 countries, Japan was ranked second in suicide prevalence (24.4 suicides per 100,000 people), following Russia [1]. During the last decade, the incidence of reported suicide rose dramatically to over 30,000 and has continued at this high rate [2]. In May 2010, the National Police Agency announced that the number of suicides in 2009 was 32,845[3], which was 596 (1.8%) more than the number in 2008. The age-stratified suicide rate (number of people who committed a suicide per 100,000 people) showed that the increase occurred in the population aged 20-60 years old, which comprises the working population. In these age groups, the major cause of suicide was health-related problems, 44% of which were accounted for by depression. Therefore, suicide and depression are closely related and need to be dealt with simultaneously.

In this paper, the problem of suicide in the occupational field will be covered in the following three topics: first, an evaluation of suicide among workers by using the mind body medicine equation hypothesis (allostatic loading hypothesis), one of the major hypotheses in psychosomatic medicine; second, a classification of the problem of suicide by metrics analysis, in which I particularly propose the adaptability of the risk assessment system to mental health problems; third, the remaining problems afflicting individuals in the occupational fields in Japan.

### Mind body medicine equation hypothesis (allostatic loading hypothesis)

Allostasis is the process of maintaining stability through change [4]. By contrast, homeostasis is the process of maintaining static equilibrium. In order to remain healthy, our bodies need to adapt to the changing environment; this adaptation is brought about by our physiological systems, which involve the body and mind. Both the body and mind are involved in response to stress, so exposure to too much stress would have an effect on both. Overwhelming stress challenges the brain's ability to maintain allostasis; it leads to metabolic wear and tear in the form of oxidative stress, which is called allostatic loading. Researchers are beginning to realize the importance of allostatic loading with regard to establishing a population-based approach in promoting health and preventing illness in occupational fields.

**Figure 1:**
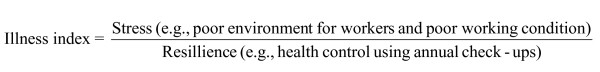
**Application of the 3 main factors in work to the Illness index**.

George Albee built a very simple model based on an earlier work in medical sociology [4]. Considering stress or allostatic loading as the numerator and resiliency to stress as the denominator, we can obtain an approximation of one's vulnerability to illness. This formula is called an illness index (Figure 1), and it would be applicable to every situation. Therefore, I believe that all healthcare personnel in occupational fields should consider this equation when evaluating the health of workers and that they should keep this equation in mind when creating prevention/treatment plans.

### Versatility of the mind body medicine equation hypothesis

When the mind body medicine equation hypothesis is applied to suicide problems in the occupational fields, the following 3 factors should be considered: (1) working environment, (2) working condition, and (3) workers' health [5]. These 3 factors, which were chosen in this paper, were based on (1) the concept of risk control called *San-kanri*, which has been traditionally used in Japanese companies [6], and on (2) the causation concepts of host, agent, and environment [7,8]. Let us apply these 3 factors (working environment, working condition, and workers' health) to the equation.

Although "working environment" and "working condition" can be assigned to the numerator or denominator, they should be allocated to the denominator in order to lower the illness index. In Figure 1, "poor environment for workers" and "poor working conditions" have been allocated to the numerator, since these factors are assumed to be problems. "Working environment" and "working condition" are issues that we can change and deal with. "Workers' health" seems to be a relatively vague concept, but in this paper, I discuss it in the light of health control for workers. Annual health check-ups are undertaken for health control among workers in Japan. Health check-up is a "good" method to find unhealthy workers; therefore, it should be allocated to the denominator.

### Matrix analysis for suicide problems in occupational fields

Matrix analysis is one of the major strategies to tackle problems in occupational fields. Haddon used this matrix analysis in occupational injury prevention, which is based on the concept that injury events can be broken down into pre-injury, injury, and post-injury phases [8]. This concept is also combined with the traditional causation concepts mentioned above, resulting in a method for breaking down an injury situation and thinking about possible interventions. Before performing matrix analysis, we need to decide the unit of measurement on the X and Y axes of the matrix table. In this matrix analysis, I used the X axis to represent the three prevention levels--;primary, secondary, and tertiary--;and the Y axis to represent the aforementioned three factors--;working environment, working condition, and workers' health (Table 1).

**Table 1:** Matrix assessment for suicide prevention in occupational fields

	Primary prevention	Secondary prevention	Tertiary prevention
**Working environment**	Health education Hiyari-Hat activity Workplace inspection	Risk assessment	Improvement of working environment

**Working condition**	Quality/Quantity control of work	The same as the primary prevention	The same as the primary prevention

**Workers' health**	Early detection of sleep disorders	Early detection of depression	Suicide prevention

### Management of working environment

#### Primary prevention in the working environment

One of the best ways to reduce suicide risk would be education for workers about mental health, including education about suicide with the help of real or simulated cases. Mentally ill people face a lot of social problems related to their ill health, as well as psychological problems. It is therefore difficult to spread awareness about mental problems, including background information related to the disease, among workers. The use of textbooks or PowerPoint presentations is not feasible for communicating this information to workers. However, a teaching style using examples of real or simulated cases is vivid and understandable even in the case of recipients who are not mentally ill. This teaching style is already used at some medical schools in both Japan and the U.S [6].

Traditionally, case-based teaching uses short cases such as vignettes or examples of a principle or concept that an instructor wishes to illustrate. Paul Lawrence, one of the founding fathers of the Harvard Business School, described case-style teaching as follows: "A good case is the vehicle by which a chunk of reality is brought into the classroom to be worked over by the class and the instructor" [9]. The advantage of case method study is that by studying practical cases, workers can learn about multidimensional problems involving various aspects such as the utilization of knowledge in their actions, general understanding of the patients' background and motivation, and ethical matters by smoothing the relationship between medicine and social issues [10]. These relationships were of paramount importance particularly in the field of mental health.

In addition to the education for workers, I recommend two strategies to avert suicide at this primary level: broadening of the concept of "*Hiyari-Hat *activity" and "Routine inspections by professionals" in the mental health field in companies. Hiyari-Hat activity is an activity based on Heinrich's law [11] in which workers report incidents they experienced however insignificant it may be. This is because Heinrich, the grandfather of injury prevention, mentioned that in a workplace, for every accident that causes a major injury, there are 29 accidents that cause minor injuries and 330 accidents that cause no injuries [11]. Nowadays, professionals of industrial hygiene deal with not only physical injury but also mental injury. The danger of mental trauma in workplaces definitely exists; places where the boss is known to be bitter or swaggering might have a history of a high resignation rate or high levels of depression among staff compared to other workplaces. Therefore, Hiyari-Hat activity should implemented in workplaces of such repute. Furthermore, for the same reason, routine inspections by professionals should also be implemented at such workplaces [5].

Mental health awareness, Hiyari-Hat activity, and routine inspection are recommended as primary prevention measures in the working environment.

#### Secondary prevention in the working environment

**Figure 2:**
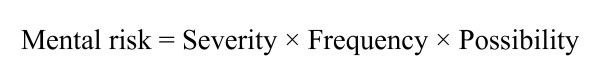
**Formula for estimating mental risk in occupational health**.

As a secondary prevention measure, I propose that risk assessment analysis be extended to the mental health field. Many Japanese companies have started establishing risk management systems since 2006, following the passing of the revised Industrial Safety and Health Act, which made the implementation of a risk management system mandatory [12]. Although there is no consensus on the ideal management model, I propose that mental risks also be evaluated by a risk management system for injury prevention. Figure 2 shows a suggested formula based on multiplication of the following three factors: (1) severity, (2) frequency, and (3) possibility [5]. First, these three factors should be scored individually, and then the multiplied score should be evaluated. The concrete process and recommended level of scores are shown in Tables 2, 3, 4 and 5. Through this process, it can be determined whether a workplace environment requires reformation. However, there are no standard scores because these scores will differ across occupational fields. For example, let us consider an incident wherein a man was suspended because he suffered from depression. The cause of his depression was severe bullying by his boss on a daily basis (we presume there was a causal relationship between the bullying and his depression). This particular boss had a history of bullying behavior with many subordinates, over 50% of whom had reported feeling depressed on account of this behavior. In this case, the risk is calculated as follows: [7 (suspended) × 5 (everyday) × 10 (happened to over 50% of employees) = 350]. According to Table 5, a score of 350 implies "need for urgent reform" for that environment, which probably means that the boss needs to be reformed. Thus, as a secondary prevention measure, evaluating the working condition by means of a risk management system is recommended.

**Table 2:** Scored severity of mental disorders and benchmarks

Severity		
**Score**	**Severity of disorders**	**Concrete benchmark**

1	Mild	Fixed by the individual himself/herself
5	No cessation	Need for physician
7	Cessation	Need for hospitalization
10	Permanent complications	Death or permanent disability

**Table 3:** Scored frequency of mental disorders and benchmarks

Frequency		
**Score**	**Frequency of occurrence**	**Concrete benchmark**

1	Rare	Once or more/year
2	Hardly ever	Once or more/6 months
3	Often	Once/month
4	Usually	Once/week
5	Always	Once/day

**Table 4:** Scored possibility of mental disorders and benchmarks

Possibility		
**Score**	**Possibility of occurrence**	**Concrete benchmark**

1	Rare	Rarely happens
5	Possible	Below 50%
10	Probable	Over 50%
20	Always	Every time

**Table 5:** Risk assessment of mental problems and determination of the need for reformation in the workplace environment

Risk assessment		
**Multiplied scores**	**Determination of outcome**	**Judgment of necessity**

401-800	Not acceptable	Need for emergent reform
201-400	Severe risk	Need for urgent reform
101-200	Moderate risk	Need for reform
51-100	Mild risk	Need for reevaluation
1-50	Acceptable	Status quo

#### Tertiary prevention in the working environment

A tertiary level measure would mainly involve ensuring that mentally ill workers who are given therapy do not suffer a relapse; in other words, it would involve re-evaluation of treated workers and improvement of their working environment. In addition to this, regular inspection by occupational physicians or industrial hygiene professionals would be necessary. From the viewpoint of improving the work environment, conversion of work-position or management of interpersonal relationships would be needed; however it would be the responsibility of the personnel section.

### Management of work (primary, secondary, and tertiary prevention in the working environment)

I suppose that preventive activity as a part of work management has already been undertaken by many companies because people are instinctively aware of its effect on the quality and quantity of work. For example, in the case of an overworked worker, his/her workload is reduced. In the case of a worker who does not receive enough support, the supervisor or co-worker provides him/her with support. Academically, this control is divided into demand-control and support, by the famous Job Contents Questionnaire (JCQ) [13,14]. The JCQ includes 22 questions constituting a minimum set of questions for the following four major scales: (1) decision latitude with decision authority and skill discretion, (2) psychological demand, (3) supervisor support, and (4) coworker support. We can evaluate the working condition by the Japanese version of the JCQ [15]. After the assessment of workers with the questionnaire, each scale--;low control, excessive pressure by the boss, low skill, high psychological demand, and low support by both the supervisor and coworkers--;should be reevaluated and ameliorated. Basically, work is a painful activity per se compared to playing, so an appropriate load that would not impair the mental health of workers is ideal.

The other famous model usually applied to evaluate workers is the effort-reward imbalance (ERI) model, which claims that failed reciprocity in terms of high efforts spent and low rewards received in turn is likely to elicit stress responses in exposed people [16,17]. Conversely, reduced stress evoked by appropriate rewards promote healthy conditions. According to the model, effort at work is spent as part of a social contract that reciprocates effort by adequate reward. Rewards are distributed by three transmitter systems: money, esteem, and career opportunities. Although each one of these components of work-related rewards was shown to matter for health, I put my emphasis on esteem because money and career opportunities are not flexible. I believe that if supervisors have the right attitude and provide positive feedback or appropriate rewards to deserving workers, it would lubricate their relationship and increase work efficiency.

### Management of health

#### Primary prevention in terms of workers' health

Most studies on tackling depression and suicide have dealt with primary prevention in terms of the health of workers. Some proposals have already been implemented by the leadership of the Cabinet Office in 2010 [18], and some are under discussion by the Ministry of Health, Labour and Welfare [19].

**Figure 3:**
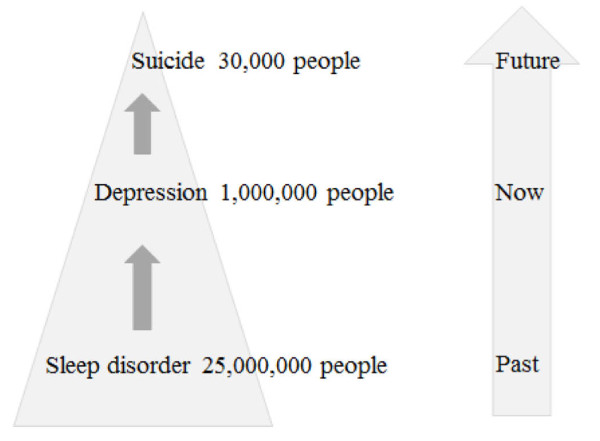
**Relationships between suicide, depression, and sleep disorders with regard to the annual number of individuals and time (A schema of management of workers' health)**.

We took up the challenge of reducing the incidence of depression and suicide in Japan in 2006, by our publication in the WHO policy paper [2]. In this paper, we showed the current condition of depression and suicide in Japan and suggested a possible strategy to avert these diseases. The schema of the health management strategy we proposed is shown in Figure 3, which shows three points of interventions and chronological relationships. As a primary prevention measure for suicide related to workers' health, sleep disorders should be targeted because these disorders are a symptom of depression and indicative of potential suicide [20]. Our study showed that the future risk of depression was high among people with sleep problems (Table 6) [21]. Thus, if sleep disorders are treated appropriately, it would dramatically decrease the risk of depression and suicide. In fact, the National Police Agency reported that the number of suicides as of March in 2010, when the sleep campaign to decrease suicide was implemented, was decreased compared to that in 2009 [22].

**Table 6:** Symptoms indicative of future depression (revised table in ref 13)

Selected SDS items in 1985	Odds ratio [95% CI]
Somatic	
Sleep disturbance	2.3 [1.1-4.9]
Constipation	1.7 [0.9-3.1]
Fatigue	2.1 [1.1-3.9]
Psychological	
Confusion	1.2 [0.8-1.8]
Psychological agitation	2.0 [0.9-4.3]
Irritability	1.6 [0.8-3.2]

Note: Logistic regression analysis was performed to select related Self-rating Depression Scale (SDS) items in 1985 for future depressive state. Six items were selected in Spearman's analyses. See ref. [13] for details.

As a concrete tactic, I recommend the following three strategies to find workers with sleep problems: (1) a screening questionnaire that includes questions on sleep timings and patterns, (2) strict evaluation of overworked workers, and (3) provision of information about sleep. The screening questionnaire is useful for finding potentially problematic workers with sleep problems. Poor sleep impairs mental health. Although the questionnaire is based on subjective declaration, if particularly problematic people are identified from the questionnaire, a scrutinizing interview should be scheduled to evaluate the problem more precisely. Overworked people should be carefully evaluated. Overwork is directly related to sleep deprivation, because if people work an additional two hours (perhaps a total of 10 working hours), they have only 14 hours left in a day. These 14 hours are used up in commuting, eating, and doing household chores, apart from sleeping. For people who overwork regularly, an additional two hours of work should warrant caution, and an additional four hours of work should be considered dangerous [23].

Furthermore, providing workers with correct information about sleep is essential; this could be done by asking them to attend professional lectures on sleep. Many people are not properly aware of the importance of sleep, so even a basic lecture about sleep would be useful.

#### Secondary prevention in terms of worker health

In this section, the main strategy is depression screening. Depression is one of the major causes of suicide. Over 60% of individuals who commit suicide have been identified as suffering from depression [24]. To reduce the number of suicide attempts, it is first necessary to identify people who are depressed or who could become depressed [24,25]. Although screening interviews based on the full criteria of the Statistical Manual of Mental Disorders, 4th edition (DSM-IV), are ideal, a simpler method is required to promote screening for depression nationwide. Our previous study showed that a one-item screening question (depressed feeling) in women and double-item question (depressed feeling and feeling miserable) proved to be adequate to screen depression in occupational fields [25]. The use of questionnaires followed by interviews would be the most feasible strategy for depression screening.

#### Tertiary prevention in terms of worker health

Tactics at this level would be based on suicide per se, targeting those who have a history of mental disease, particularly depression and suicide ideation. However, until recently, it has been considered taboo to talk about suicide problems in Japan, because most Japanese are biased against suicide. It was therefore difficult to establish strategies for prevention based on suicide itself in occupational fields. It would be an occupational hazard for a physician to ask questions about suicide ideation to workers, because such questions would be considered radical and inappropriate, perhaps leading their supervisor to disallow such questionnaires or interviews. The situation in Japan is however gradually changing, with the mass media spreading awareness about suicide. Nowadays, it therefore may be possible to freely talk about mental health including suicide ideation with workers. Particularly people who have a history of mental problems should be regularly evaluated via interviews. Since the number of such people is rather small, it would be possible to implement this strategy to prevent suicide at the tertiary level.

Suicide prevention by identifying people with suicide ideation by using questionnaires and interviews, particularly in the case of the target group, is a good tactic at this level.

## Conclusions

The mind body medicine equation would be applicable to depression and suicide prevention in occupational fields, considering the working environment, working condition, and workers' health. Matrix analysis is one of the appropriate tactics for suicide prevention because it would allow for the technical evaluation of suicide as a tangible problem. Mental health problems, including suicide, are rather tricky to deal with, because it involves the study of individual cases. However, the matrix analysis enables us to view mental problems as tangible problems akin to infectious diseases and could show us the limitations of our ongoing plan for tackling mental health problems among workers.

The main limitation is difficulty in recruiting the correct individuals for this treatment since it involves sensitive personal issues. A full-time nurse or occupational physician would be appropriate for dealing with confidential information, but a worker who is temporarily recruited would not be as efficient as a health professional. I do not think matrix analysis and broadening the concept of risk analysis to mental health are the answers to all the problems in occupational fields. However, Japan has preeminent methods for health control for workers, mandatory annual health check-ups, and risk management. Used in combination, these methods are most feasible for tackling suicide problems in occupational fields, and we should aim to decrease the number of annual suicides to less than 30,000 in the future.

## Competing interests

The author declares that they have no competing interests.

## Authors' contributions

The author wrote the manuscript and holds final responsibility for the decision to submit the manuscript for publication.
